# One-Pot Synthesis of Chlorophyll-Assisted Exfoliated MoS_2_/WS_2_ Heterostructures via Liquid-Phase Exfoliation Method for Photocatalytic Hydrogen Production

**DOI:** 10.3390/nano11092436

**Published:** 2021-09-18

**Authors:** I-Wen P. Chen, Yan-Ming Lai, Wei-Sheng Liao

**Affiliations:** Department of Applied Science, National Taitung University, 369, Sec. 2, University Rd., Taitung City 95092, Taiwan; timtim355798@gmail.com (Y.-M.L.); kyle3261010@gmail.com (W.-S.L.)

**Keywords:** liquid-phase exfoliation, MoS_2_, WS_2_, heterostructure, chlorophyll, photoelectrochemical

## Abstract

Developing strategies for producing hydrogen economically and in greener ways is still an unaccomplished goal. Photoelectrochemical (PEC) water splitting using photoelectrodes under neutral electrolyte conditions provides possibly one of the greenest routes to produce hydrogen. Here, we demonstrate that chlorophyll extracts can be used as an efficient exfoliant to exfoliate bulk MoS_2_ and WS_2_ to form a thin layer of a MoS_2_/WS_2_ heterostructure. Thin films of solution-processed MoS_2_ and WS_2_ nanosheets display photocurrent densities of −1 and −5 mA/cm^2^, respectively, and hydrogen evolution under simulated solar irradiation. The exfoliated WS_2_ is significantly more efficient than the exfoliated MoS_2_; however, the MoS_2_/WS_2_ heterostructure results in a 2500% increase in photocurrent densities compared to the individual constituents and over 12 h of PEC durability under a neutral electrolyte. Surprisingly, in real seawater, the MoS_2_/WS_2_ heterostructure exhibits stable hydrogen production after solar illumination for 12 h. The synthesis method showed, for the first time, how the MoS_2_/WS_2_ heterostructure can be used to produce hydrogen effectively. Our findings highlight the prospects for this heterostructure, which could be coupled with various processes towards improving PEC efficiency and applications.

## 1. Introduction

The origin of the industrial revolution has created the convenience of human life and the development of science and technology. People’s demand for energy has increased year by year, and the massive energy supplies are from fossil fuels, which lead to environmental pollution and extreme climate change. Hence, a clean and carbon-free energy source should be developed to preserve the environment [1,2]. Therefore, the development of environmentally friendly, high-efficiency, and sustainable energy resources is an unaccomplished goal. Hydrogen fuel has long been regarded as a substitute for fossil fuels and has the advantage of being a clean and sustainable energy source [3]. Since the electrolysis of water requires a lot of electric energy, the energy released by the combustion of water-splitting-generated hydrogen will not be equal to the consumed electric energy. Solar energy is generally regarded as a free, abundant, and continuously renewable clean energy source that can fulfill future human energy needs. Therefore, solar-driven photoelectrochemical (PEC) water splitting is regarded as an alternative method and offers promising approaches to convert solar energy into storable and environmentally friendly hydrogen fuel.

After the first report of the Honda–Fujishima effect [4], Honda et al. demonstrated that the electrochemical photolysis of water can be achieved by utilizing semiconductor-based materials. However, the materials absorb solar energy to induce water splitting to generate H_2_ fuel, which was limited by the following factors: (1) poor absorption in the visible region, (2) fast electron–hole recombination, and (3) limited active sites [5]. To overcome these bottlenecks, several strategies such as co-catalysts [6,7,8], band gap engineering [9,10], and the construction of a heterojunction [11] were proposed. Therefore, researchers have been committed to designing semiconductor-based materials with a variety of structural morphologies to enhance photolysis performances for a variety of photocatalytic applications. 

Two-dimensional transition metal dichalcogenide (TMD) layered materials have recently attracted renewed interest because of their superior light–matter interactions. These interactions originate from their intrinsic two-dimensionality, d-electron orbital character, and anisotropic structure. Usually, they absorb 5−10% of incident light in the visible range, and the exfoliated thin sheets have shown photovoltaic characteristics. A range of optoelectronic devices predominantly based on thin sheets have been demonstrated [12,13,14]. The pristine two-dimensional thin sheet results in a direct band gap in the visible range, such as ∼2.0 eV for tungsten disulfide (WS_2_) and ∼1.8 eV for molybdenum disulfide (MoS_2_) [15], while in bulk form, they retain an indirect band gap of between 1.1 and 1.4 eV. These two materials have the same crystal structure and present a similar electronic band structure. According to computational studies [16], the valence band position of MoS_2_ and WS_2_ thin sheets is more positive than the water oxidation potential (1.23 V vs. standard hydrogen electrode (SHE)) [17,18]. However, their bulk forms do not achieve the thermodynamic criteria for PEC water splitting. Hence, the use of two-dimensional (2D) thin sheets as photocatalysts would have great advantages over bulk form materials, such as increased specific active sites and the lack of crystallographic defects on the surface. Moreover, the versatility of the 2D thin sheet materials offered by the deposition from the liquid phase suspension can facilitate the fabrication of novel heterojunction systems [19]. Here, we report a one-pot synthesis of the exfoliated MoS_2_/WS_2_ thin sheet heterostructure by using the exfoliant of chlorophyll extracts with the liquid-phase exfoliation (LPE) method. Chlorophyll is a major pigment used in natural photosynthesis and is one of nature’s abundant materials on earth. The molecular structure of chlorophyll is a conjugated π-electronic structure. Owing to the π–π interaction, it could spontaneously lie parallel on a flat surface such as graphene and TMDs [20]. The heterostructure exhibits a photocurrent density 2500% greater than that of films at zero potential comprised of the individual constituents. This is attributed to the highly efficient exciton dissociation generated by the creation of MoS_2_/WS_2_ heterostructures [21]. The charge-separated states in the heterostructures have also been envisioned to be long-lived, even though the close distance of the generated holes and electrons increases the possibility of water splitting occurring [22]. The stability of the exfoliated MoS_2_/WS_2_ thin sheet heterostructure for more than 12 h was achieved in a neutral electrolyte. Moreover, in real seawater, the exfoliated MoS_2_/WS_2_ thin sheet heterostructure exhibits stable performance after continuous visible light illumination for 12 h. We believe that our findings will inspire the further development of novel heterostructure strategies that will provide the ability to enhance PEC performance and long-term stability for a multitude of 2D materials.

## 2. Materials and Methods

**Chemicals.** Molybdenum(IV) sulfide (MoS_2_, 99% metal basis, ∼325 mesh powder) and WS_2_ powder (99% metal basis; ∼325 mesh powder) were used. All solvents were analytical grade and used as received without further purification.

**Preparation of extracted chlorophylls.** Sapium sebiferum leaves (40 g) were ground by a pestle and mortar. Then, the smashed leaf powders were transferred to a 1 L beaker, and 1 L acetone was poured into the beaker. After stirring at 800 rpm for 8 h, the extracted chlorophylls were filtered through a polyvinylidene difluoride (PVDF) membrane (0.22 mm) to remove impurities. The filtered solution of the extracted chlorophylls was centrifuged at 3000 rpm for 1 h, and the precipitate was discarded. The solution concentration of the extracted chlorophylls was ∼5 mg/L. The sample was stored at −20 °C.

**Synthesis of MoS_2_/WS_2_ nanosheet suspension.** A total of 0.2 g WS_2_ and 0.2 g MoS_2_ were put in a double water jacket (passing 4 °C circulating cooling water), and 150 mL acetone and 0.64 mL chlorophyll extract were added. Then, an ultrasonic cell grinder (power 100 W) was used for 2 h, with 2 s of rest for every 10 s of sonication. After sonication, a dark green suspension was obtained, which was poured into a serum bottle for storage. 

**Preparation of MoS_2_/WS_2_ nanosheet composite electrode**. The FTO (Florine Doped Tin Oxide) conductive glass (1 cm × 2 cm) was cleaned and placed into a glass bottle. Acetone was added, and a bath ultrasonic treatment was performed for 30 min. The prepared suspension solution was dropped (drop 100, 200, 300, ..., 800 μL, respectively) onto the conductive surface of the FTO conductive glass and baked at 180 °C for 30 min. The sample was cooled to room temperature for the electrochemical experiments. Figure S1 shows the representative morphology of MoS_2_/WS_2_ 1:1 on FTO. 

**Characterization**. The structural properties of the prepared materials were characterized by using a UV–Vis spectrometer (U-2900; Hitachi, Tokyo, Japan), fiber-coupled Raman spectrometer (532 nm; Horiba Jobin Yvon, Kyoto, Japan), X-ray diffraction (XRD; Bruker AXS D8 Advance, Karlsruhe, Germany), X-ray photoelectron spectroscopy (XPS; Thermo K-Alpha, Waltham, MA, USA), and JEOL Hitachi H-7100 transmission electron microscopy (TEM; Hitachi, Tokyo, Japan). Lifetime spectra of the materials were measured using a pulsed diode laser as an excitation source with a central emission wavelength of 375 nm (LDH-P-C-375B; PicoQuant GmbH, Berlin, Germany) and a photoluminescence spectrophotometer (PL; Hitachi F-7000, Tokyo, Japan). The ultrasonicator (Q700; Qsonica, Newtown, CT, USA) was utilized to exfoliate bulk WS_2_ to become thin sheets and to prepare a heterostructure of MoS_2_/WS_2_. The electrochemical properties were tested using the electrochemical workstation CHI 7927E (CH Instruments Inc., Austin, TX, USA). In this three-electrode system, the materials, Ag/AgCl, and the graphite rod act as a working electrode, reference electrode, and counter electrode, respectively. Electrochemical impedance spectroscopy (EIS, CH Instruments Inc., Austin, TX, USA) was carried out from 1 Hz to 1000 kHz at 0.65 V potential.

## 3. Results and Discussions

**Figure 1:**
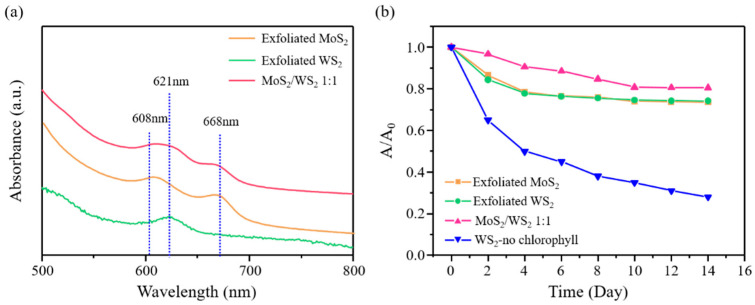
(**a**) UV–Vis spectra, (**b**) comparative stability of chlorophyll-assisted exfoliated MoS_2_, WS_2_, and mixed MoS_2_/WS_2_ suspensions.

To thoroughly characterize the chlorophyll-assisted exfoliated MoS_2_/WS_2_ heterostructures, both qualitative and quantitative characterizations are required. Figure 1a shows the optical absorbance spectra of the exfoliated TMD thin sheet. The two fully resolved absorbance bands at 608 and 668 nm resemble those results from a mechanically exfoliated MoS_2_ monolayer, which shows that the solution exfoliation method offers the presence of a large amount of exfoliated monolayer structures [23,24]. In addition, the exfoliated WS_2_ suspension shows an absorbance band at 621 nm, which indicates that the exfoliated WS_2_ sheets in the dispersions were close to the monolayer [25,26]. Additionally, the mixed bulk MoS_2_ and WS_2_ powder can be successfully exfoliated to thin sheets [27], and the exfoliated MoS_2_/WS_2_ suspension shows the characteristic absorbance bands of the monolayer MoS_2_ and WS_2_, as shown in Figure 1a (reddish line). Figure 1b shows no significant decay in the absorbance of the chlorophyll-assisted exfoliated mixed thin sheet suspension, indicating the superior stability of the chlorophyll-assisted liquid-phase exfoliation method in the scalable production of TMDs. 

**Figure 2:**
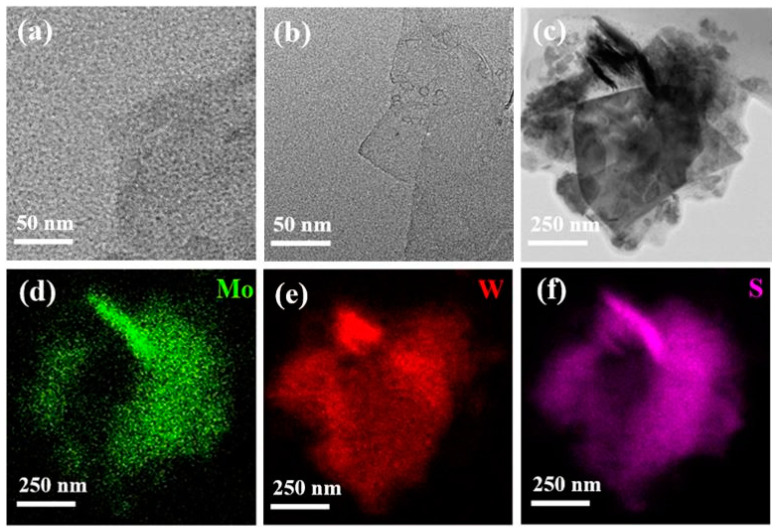
TEM images of (**a**) MoS_2_, (**b**) WS_2_, (**c**) MoS_2_/WS_2_ 1:1; (**d**–**f**) EDS mapping images of MoS_2_/WS_2_.

The TEM images (Figure 2a,b) show that the bulk MoS_2_ and the bulk WS_2_ were exfoliated into thin sheets with the assistance of the extracted chlorophylls. Figure S2 shows the TEM image of the bulk MoS_2_ material. Figure 2c shows the TEM image of the exfoliated MoS_2_/WS_2_ suspension. Figure 2d–f show the energy dispersive spectroscopy (EDS) characterization of the MoS_2_/WS_2_ heterostructure. The Mo and W signals are mixed together, confirming that the exfoliated MoS_2_ and WS_2_ thin sheets can be homogeneously distributed in the MoS_2_/WS_2_ heterostructure via a one-pot liquid phase synthesis method. 

**Figure 3:**
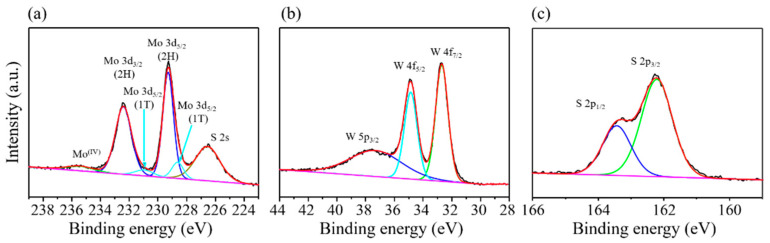
XPS spectra of the MoS_2_/WS_2_ heterostructure. (**a**) Mo 3d, (**b**) W 4f, and (**c**) S 2p.

To understand the chemical composition of the MoS_2_/WS_2_ heterostructure, XPS was utilized to analyze the chemical properties of Mo, W, and S. Figure 3a shows the Mo 3d spectrum on the sample of the MoS_2_/WS_2_ heterostructure. The Mo 3d spectrum shows peaks centered at 229.3 and 232.4 eV, representing the Mo^4+^ 3d_5/2_ and Mo^4+^ 3d_3/2_ components of the semiconducting type MoS_2_. The other peaks at 228.6 and 231.4 eV are attributed to the Mo^4+^ 3d_5/2_ and Mo^4+^ 3d_3/2_ components of the metallic type MoS_2_, respectively. Figure 3b shows the peaks at 32.7 eV, 34.9 eV, and 37.6 eV, which correspond to the W 4f_7/2_, W 4f_5/2_, and W 5p_3/2_ components, respectively. Similarly, in the S 2p core-level XPS spectrum, Figure 3c shows two peaks at 162.2eV and 163.4 eV in the MoS_2_/WS_2_ heterostructure sample, indicating that the S atoms retain their pristine property [28,29,30,31]. These results are superior to those of electrochemically exfoliated TMD sheets that oxidize the sulfur to form sulfur oxides.

**Figure 4:**
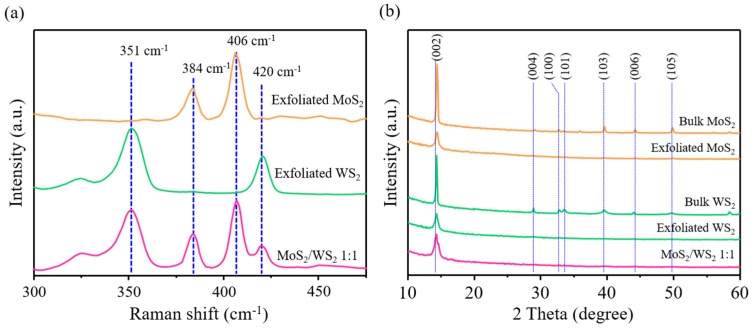
(**a**) Raman and (**b**) XRD spectra of the exfoliated MoS_2_, WS_2_, and MoS_2_/WS_2_ 1:1 thin sheets.

To understand the intrinsic properties of the exfoliated MoS_2_ and WS_2_ thin sheets in the MoS_2_/WS_2_ heterostructure, a non-destructive Raman spectroscopy technique was used. Figure 4 shows the Raman spectra of the exfoliated MoS_2_, WS_2_, and MoS_2_/WS_2_. For the exfoliated MoS_2_ thin sheets, two peaks can be observed at 384 cm^−1^ and 406 cm^−1^ corresponding to E^1^_2g_ and A_1g_, respectively. The measured difference between the two modes of the exfoliated MoS_2_ thin sheets is 22 cm^−1^, indicating that the monolayer MoS_2_ structure was successfully prepared [32]. For the exfoliated WS_2_ thin sheets, the E^1^_2g_ and A_1g_ values are 351 cm^−1^ and 420 cm^−1^ [33], respectively. The intensity of E^1^_2g_ is two times higher than that of A_1g_, indicating that the thickness of the exfoliated WS_2_ sheets remains a monolayer structure [33]. Moreover, the Raman spectrum of the MoS_2_/WS_2_ heterostructure shows a profile identical to the exfoliated MoS_2_ and WS_2_ thin sheets, which further supports our proposed one-pot synthesis method for successfully preparing the MoS_2_/WS_2_ heterostructure. Figure 4b shows the XRD analysis of the bulk form and phase of exfoliated materials of MoS_2_, WS_2_, and the MoS_2_/WS_2_ heterostructure. The bulk MoS_2_ and bulk WS_2_ show very strong (002) peaks at the diffraction angle of 2*θ* = 14.4°, and the subsequent peaks of 29°, 32.6°, 33.5°, 39.5°, 44.2°, 49.8°, and 58.4° correspond to the crystal planes of (004), (100), (101), (103), (006), (105), and (008), respectively [34,35,36]. After exfoliating the bulk TMDs to achieve thin sheets, the full width at half maximum (FWHM) of the (002) peak of the exfoliated MoS_2_ and WS_2_ thin sheets is broader than the bulk form, demonstrating the formation of TMD thin sheets [36]. Moreover, the XRD spectrum of the MoS_2_/WS_2_ heterostructure shows the same FWHM of the (002) peak of the exfoliated MoS_2_ and WS_2_ thin sheets, indicating that the exfoliated MoS_2_ and WS_2_ will not reaggregate to the bulk form.

**Figure 5:**
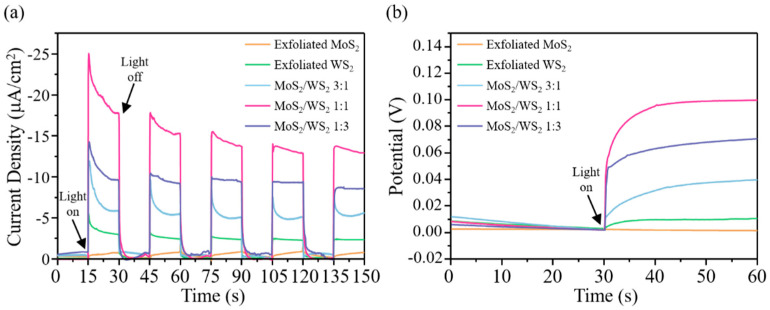
(**a**) Transient photocurrent and (**b**) Open circuit potential electrochemical test of different samples. Light was turned on at 30 s.

The PEC activity for the hydrogen evolution of MoS_2_ and WS_2_ thin sheets and their MoS_2_/WS_2_ heterostructure was studied. The PEC properties were carried out in a complete PEC cell using an aqueous electrolyte under neutral conditions. Na_2_SO_4_ was utilized as the electrolyte because it does not interact or interfere with most of the electrode or electrochemical reactions, respectively. Besides, the Na_2_SO_4_ electrolyte provides no environment for H^+^ generation. Hence, the actual water splitting performance of the materials can be determined. Figure S3 shows linear sweep voltammetry (LSV) curves of the exfoliated single material and MoS_2_/WS_2_ heterostructure under dark and light conditions. MoS_2_/WS_2_ 1:1 shows the highest PEC performance compared to the single component of the exfoliated material. Chronoamperometry (CA) scans (Figure 5a) under chopped irradiation using a 250 W Xe lamp with a UV filter (420 nm) confirm the photocurrent generated by the MoS_2_, WS_2_, and MoS_2_/WS_2_ heterostructure electrodes. The applied voltage was 0 V. All three samples show an ultrafast PEC current response when the visible light source was changed between the on and off states. In the visible light-on condition, the decay of the PEC current is caused by the photogenerated electron and hole recombination. When the visible light was turned off, the photogenerated electrons and holes at the surface of the materials immediately vanished. Under the same illumination conditions, the peak PEC current density of the exfoliated MoS_2_, WS_2_, MoS_2_/WS_2_ 3:1, MoS_2_/WS_2_ 1:1, and MoS_2_/WS_2_ 1:3 is −1, −5, −12, −25, and −15 μA/cm^2^, respectively. The photocurrent of the MoS_2_/WS_2_ (1:1) electrode shows a 2500% and 500% enhancement compared to those of the exfoliated MoS_2_ and WS_2_, respectively.

To shed light on the amount of photogenerated electrons and the change in voltage, open current potential (OCP) measurements were conducted, which give information about the surface recombination. Figure 5b shows the OCP of the TMD electrode under chopped irradiation. We observe that the MoS_2_/WS_2_ 1:1 photoelectrode shows the highest change in OCP. This result demonstrates that the surface recombination between photogenerated electrons and holes in the MoS_2_/WS_2_ 1:1 heterojunction is inhibited, which indicates that much more effective charges are used to perform the water reduction reaction. Hence, the formation of a heterogeneous structure can significantly enhance the separation of electrons and holes during light irradiation.

**Figure 6:**
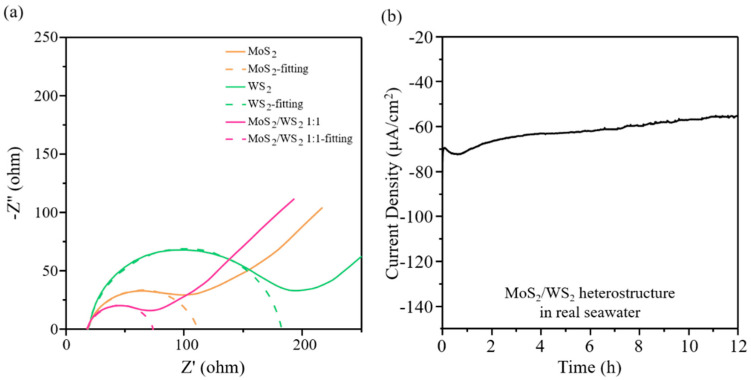
(**a**) Nyquist plots of MoS_2_, WS_2_, and MoS_2_/WS_2_ 1:1. (**b**) Irradiation time (*x*-axis) dependence of the HER for MoS_2_/WS_2_ 1:1 (at 0 V in real seawater).

To deduce the charge transport properties of the interface between the electrode and the electrolyte, electrochemical impedance spectroscopy (EIS) was performed. Figure 6a shows the Nyquist plots of MoS_2_, WS_2_, and MoS_2_/WS_2_ 1:1, which are performed under light illumination at 0 V vs. Ag/AgCl. The solid line represents the real resistance data of the sample, and the dashed line represents the charge transfer resistance (R_ct_) obtained by fitting the measured data using Z view software. The semicircle in the middle is the high-frequency region in EIS, and the resistance is dominated by charge transfer. In Figure 6a, we can observe that MoS_2_/WS_2_ 1:1 shows a smaller arc than that of MoS_2_ and WS_2_. Hence, the value of R_ct_ for the MoS_2_/WS_2_ 1:1 electrode is decreased compared to the exfoliated MoS_2_ and WS_2_, which demonstrates the highly efficient electron–hole separation. That is, the separation of photogenerated electron–hole pairs could be achieved in the MoS_2_/WS_2_ 1:1 heterojunction catalyst by transferring the charges to the surface-active sites and participating in the water reduction to generate H_2_. The photocatalytic stability of the MoS_2_/WS_2_ 1:1 catalyst was investigated by monitoring the generated current density of H_2_. As shown in Figure S4, the photocatalytic hydrogen evolution reaction (HER) experiments were performed under a Na_2_SO_4_ neutral electrolyte. A negligible difference in the H_2_ evolution current density is observed within a working period of over 12 h at 0 V under a neutral condition. However, as a single salt neutral electrolyte solution does not represent a real-world environment, the hydrogen generation performance of the prepared MoS_2_/WS_2_ 1:1 PEC catalyst was performed in a real-world sample (e.g., seawater) to demonstrate its stability behavior. On the basis of the ultimate goal of seawater electrolysis, hydrogen production is still an ongoing challenge. A critical issue is that most of the catalysts tend to decompose and/or deteriorate in a high-salinity condition, usually showing inferior performance and instability. Therefore, seawater, the most abundant environment in the world, is used to demonstrate the PEC hydrogen generation performance of the prepared MoS_2_/WS_2_ to further expand its practical application. Figure 6b shows that the prepared MoS_2_/WS_2_ heterostructure catalyst showed no more than a 15% decrease at a current density of ∼60 mA/cm^2^ after operation for 12 h, indicating the excellent photocatalytic durability of MoS_2_/WS_2_ 1:1 under a high-salinity condition.

**Figure 7:**
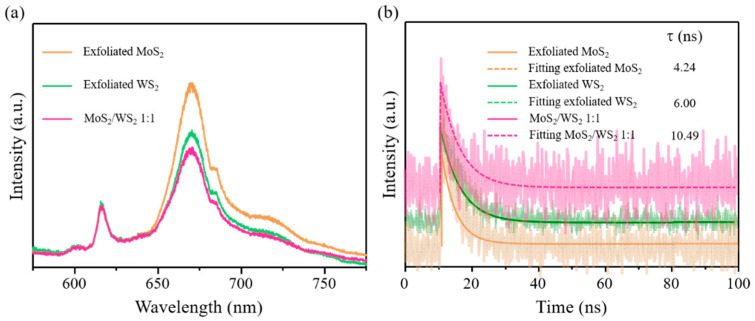
(**a**) PL spectra and (**b**) time-resolved photoluminescence studies of MoS_2_, WS_2_, and MoS_2_/WS_2_ 1:1.

To evaluate the photoinduced electron–hole pair separation capability of the materials, a PL spectroscopy study was carried out. In general, it has been widely shown that a higher PL intensity indicates a fast rate of electron–hole pair recombination, leading to inferior HER performance. Figure 7a shows the PL spectra of the as-prepared MoS_2_/WS_2_ 1:1. Obviously, the exfoliated MoS_2_ shows the highest PL intensity, indicating the fastest recombination rate of the photogenerated charges [37,38]. When MoS_2_ was mixed with WS_2_ to form the MoS_2_/WS_2_ 1:1 heterostructure, the emission intensity decreased, indicating that the photogenerated electron–hole pairs separated more efficiently. As per Figure 7b, when the heterostructure was prepared, there was an increase in the contribution of the average fluorescence quenching time. This increment indicates the evolution of new radiative pathways, which boost the transfer of a greater number of photoexcited electrons for HER. Similar observations were reported in the case of a WS_2_–BiOCl composite [39].

**Figure 8:**
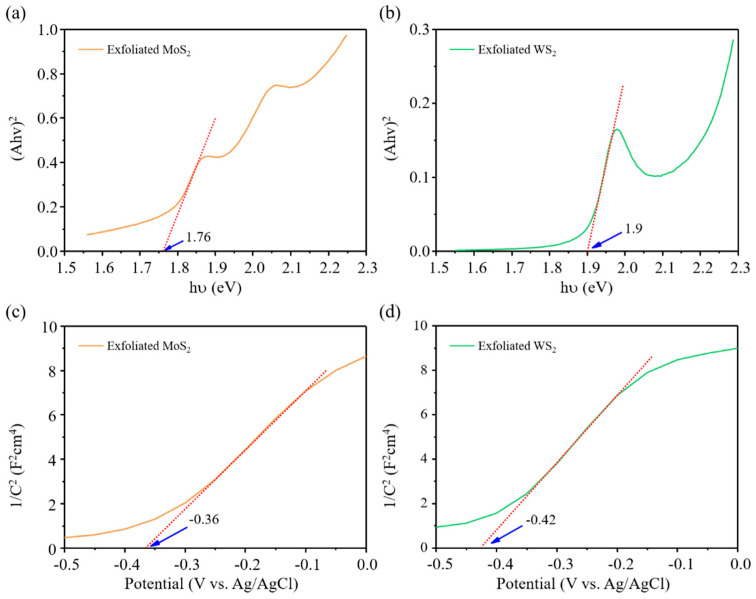
(**a,b**) Tauc and (**c,d**) Mott–Schottky plots of MoS_2_ and WS_2_.

**Figure 9:**
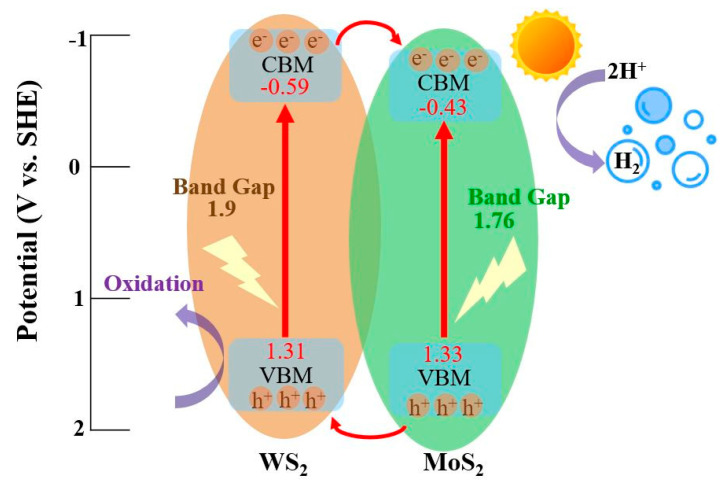
Photocatalytic mechanism of MoS_2_/WS_2_ in 0.5 M Na_2_SO_4_ (pH = 7).

In order to explore the mechanism of photocatalysis, the bandgap and conduction band position (or flat band potential (E_fb_)) of the exfoliated MoS_2_ and WS_2_ were studied. The bandgap of the materials was measured by UV–Vis spectroscopy. Figure 8a,b show that the bandgap of the exfoliated MoS_2_ and WS_2_ nanosheets is 1.76 eV and 1.9 eV, respectively. The E_fb_ of the materials was subjected to Mott–Schottky analysis under dark conditions. As shown in Figure 8c,d, the slope of the exfoliated MoS_2_ and WS_2_ was positive, indicating n-type semiconductor characteristics [40]. Generally, for n-type semiconductors, the actual E_fb_ value is 0.3 V lower than the E_fb_ value measured by the Mott–Schottky method, so the conduction band potential (relative to Ag/AgCl) of MoS_2_ and WS_2_ is −0.13 eV and −0.29 eV, respectively [25,41]. Therefore, combining the results of the bandgap and E_fb_ of the materials, the calculated valence band potentials of MoS_2_ and WS_2_ were 1.33 eV and 1.31 eV, respectively. A possible photocatalytic mechanism of the MoS_2_/WS_2_ 1:1 heterostructure is demonstrated in Figure 9. The E_fb_ of WS_2_ is more negative than the E_fb_ of MoS_2_; therefore, the electrons were forced to flow from the high-energy WS_2_ conduction band to the lower-energy MoS_2_ conduction band. At the same time, they generated holes in the valance band of MoS_2_ and WS_2_ where they are trapped by lactate ions in the solution. Therefore, the recombination between photoinduced electrons and holes can be efficiently suppressed. According to the literature report [29,42], the photocatalytic scheme of the MoS_2_/WS_2_ heterostructure could be attributed to a Type II scheme. The increase in photocatalytic activity of the MoS_2_/WS_2_ 1:1 heterostructure is due to the suitable band structure and low electron–hole recombination rate compared to the individual materials. As a consequence, more photoinduced electrons can be used for water splitting.

## 4. Conclusions

In conclusion, a simple, green, and effective one-pot synthesis was proposed to prepare the novel MoS_2_/WS_2_ heterostructure involving chlorophyll extracts as exfoliants under liquid-phase exfoliation. The exfoliant-assisted exfoliation approach provided a high-efficiency method for the scalable production of thin TMD heterostructures. The MoS_2_/WS_2_ heterostructure showed high photocatalytic performance. In order to comprehensively unveil the internal mechanism of the MoS_2_/WS_2_ heterogeneous structure, the electrochemical and optical properties of the individual MoS_2_, WS_2_, and MoS_2_/WS_2_ heterostructure were studied. The MoS_2_/WS_2_ heterostructure can split water, evolving H_2_ gas in a neutral environment under illumination with simulated sunlight. This hydrogen evolution occurs without the assistance of any co-catalysts and protection layers. The magnitude of the efficiency is 2500% higher in MoS_2_/WS_2_ heterostructure electrodes. This enhancement can be attributed to the efficient electron–hole dissociation via the band alignment across the interfaces of the two materials and, therefore, the prolongation of the photoinduced charge separation lifetime against recombination. Our results provide a solution-processable, atomically thin material system with the proper bandgap in the visible region, paving the way toward developing next-generation photocatalysts for water splitting.

## Data Availability

Not applicable.

## Supplementary Materials

The following are available online at https://www.mdpi.com/article/10.3390/nano11092436/s1. Figure S1: SEM image of the MoS_2_/WS_2_ thin film on FTO, Figure S2: TEM image of the bulk TMDs (e.g., MoS_2_), Figure S3: LSV curves of the individual materials and composite under dark (d) and light (L) conditions, Figure S4: Irradiation time (*x*-axis) dependence of the HER for MoS_2_/WS_2_ 1:1 (at 0 V in a Na_2_SO_4_ electrolyte).
