# Supplementary material for: One-Pot Synthesis of Chlorophyll-Assisted Exfoliated MoS2/WS2 Heterostructures via Liquid-Phase Exfoliation Method for Photocatalytic Hydrogen Production
Source: Nanomaterials (Basel). 2021 Sep 18;11(9):2436. doi: 10.3390/nano11092436 (PMC8469628; doi:10.3390/nano11092436)
Supplement: Supplementary file 1 [file nanomaterials-11-02436-s001.zip › nanomaterials-1381107-supplementary.pdf]

Supplementary Materials

One-Pot Synthesis of Chlorophyll-Assisted Exfoliated MoS_2/WS_2 Heterostructures via Liquid-Phase Exfoliation Method for Photocatalytic Hydrogen Production

I-Wen Peter Chen [1,*], Yan-Ming Lai [1] and Wei-Sheng Liao [1]

[1] Department of Applied Science, National Taitung University, 369, Sec. 2, University Rd., Taitung City 95092, Taiwan; timtim355798@gmail.com (Y.L.); kyle3261010@gmail.com (W.L.)
* Correspondence: iwchen@nttu.edu.tw;

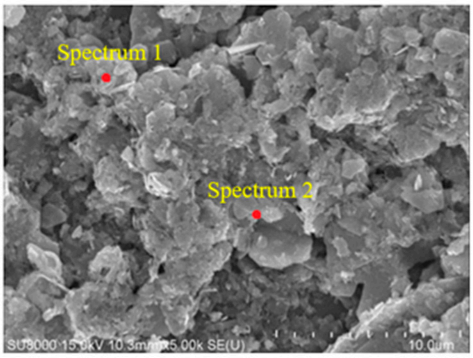

Atomic concentration [%]

Spectrum	Sulfur	Molybdenum	Tungsten
Spectrum 1	74.57	11.41	14.01
Spectrum 2	74.24	13.00	12.76

Figure S1. SEM image of the MoS_2/WS_2 thin film on FTO.

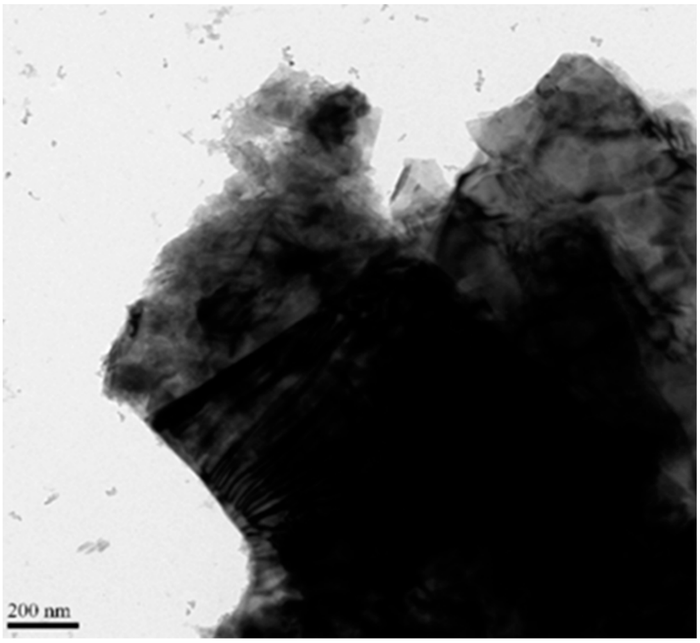

Figure S2. TEM image of the bulk TMDs (e.g. MoS_2).

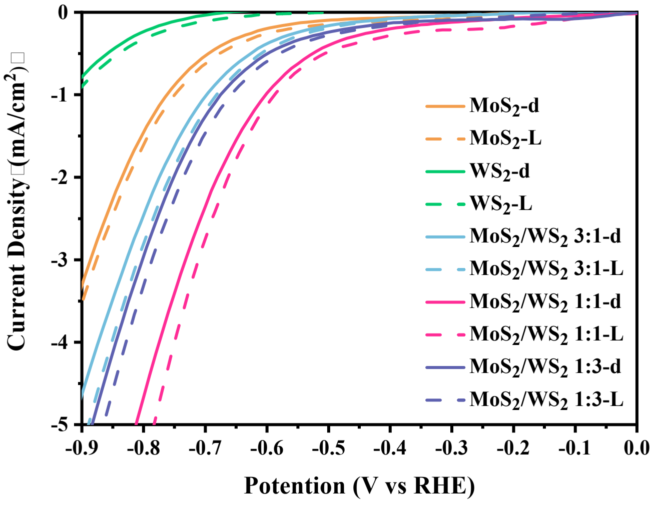

Figure S3. LSV curves of the exfoliated single material and MoS_2/WS_2 heterostructure under dark (d) and light-on (L) condition.

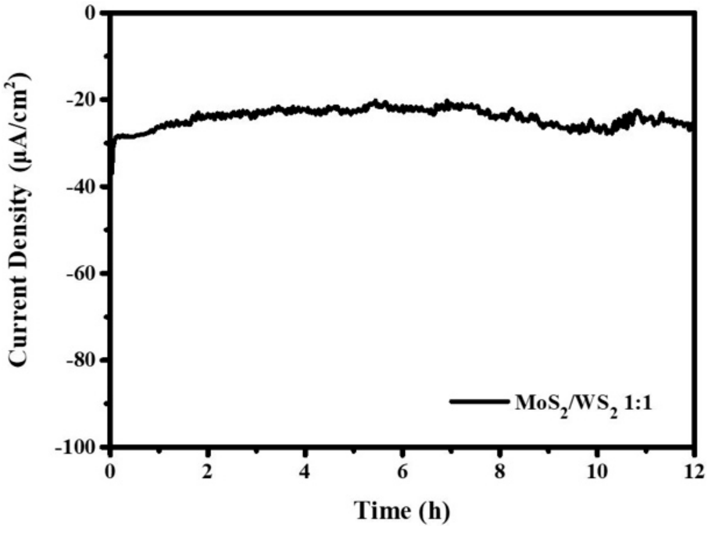

Figure S4. Irradiation time (x-axis) dependence of the HER for the MoS_2/WS_2 1:1 (at 0 V in a Na_2SO_4 electrolyte).
